# Potential Key Bases of Ribosomal RNA to Kingdom-Specific Spectra of Antibiotic Susceptibility and the Possible Archaeal Origin of Eukaryotes

**DOI:** 10.1371/journal.pone.0029468

**Published:** 2012-01-11

**Authors:** Qiang Xie, Yanhui Wang, Jinzhong Lin, Yan Qin, Ying Wang, Wenjun Bu

**Affiliations:** 1 Department of Zoology and Developmental Biology, College of Life Sciences, Nankai University, Tianjin, China; 2 National Laboratory of Biomacromolecules, Institute of Biophysics, Chinese Academy of Sciences, Beijing, China; University of South Florida College of Medicine, United States of America

## Abstract

In support of the hypothesis of the endosymbiotic origin of eukaryotes, much evidence has been found to support the idea that some organelles of eukaryotic cells originated from bacterial ancestors. Less attention has been paid to the identity of the host cell, although some biochemical and molecular genetic properties shared by archaea and eukaryotes have been documented. Through comparing 507 taxa of 16S–18S rDNA and 347 taxa of 23S–28S rDNA, we found that archaea and eukaryotes share twenty-six nucleotides signatures in ribosomal DNA. These signatures exist in all living eukaryotic organisms, whether protist, green plant, fungus, or animal. This evidence explicitly supports the archaeal origin of eukaryotes. In the ribosomal RNA, besides A2058 in *Escherichia coli* vs. G2400 in *Saccharomyces cerevisiae*, there still exist other twenties of sites, in which the bases are kingdom-specific. Some of these sites concentrate in the peptidyl transferase centre (PTC) of the 23S–28S rRNA. The results suggest potential key sites to explain the kingdom-specific spectra of drug resistance of ribosomes.

## Introduction

The parts of the theory of endosymbiosis that focus on the origins of mitochondria, plastids, and other organelles of eukaryotic cells are well known and widely accepted [1]–[4]. Meanwhile, regarding the origin of the nucleus, several different hypotheses remain hotly debated [5]. Some biochemical or molecular genetic properties that are shared by archaea and eukaryotes have been documented [6], [7], and the monophyletic group constituted by archaea and eukaryotes was named Neomura [6]. Among the shared properties of neomurans, some are well known, such as the similar ribosomal sensitivity of neomurans to antibiotics or toxins. The spectra of ribosomal sensitivity to antibiotics are associated with the structures of the ribosomes. However, due to the complexity of ribosomes, it is difficult to obtain their fine crystal structures, especially for eukaryotic ribosomes. To date, *Saccharomyces cerevisiae* and *Tetrahymena thermophila* are the sole eukaryotic species whose ribosomal structures have been determined completely [8] or partially [9] at the resolution of approximately 4 angstroms. Additionally, it is still necessary to refine the structure to a higher resolution to reduce errors [10]. The inability to achieve a higher resolution currently makes it impossible to collect abundant information on different types of eukaryotic organisms to conduct comparative studies of the three dimensional structures of ribosomes.

In addition to the difficulties with structural studies of ribosomes, it is also difficult to align the ribosomal genes (rDNA) of eukaryotes due to the intron sequences and the length variations in various regions of the rDNA [11], [12]. To date, many ribosomal signatures shared between Archaea and Bacteria have been identified [13]. The full lengths of the intron free regions of the small subunit (SSU) of eukaryotic rDNA vary from approximately 1,500 nucleotides or shorter (e.g., Fornicata, Microsporidia, *Mikrocytos* and Parabasalidea) to approximately 4,500 nucleotides or longer (e.g., Euglenida: *Distigma*). The full length of the large subunit (LSU) in eukaryotic rDNA ranges from approximately 2,500 (e.g., Microsporidia) to approximately 5,200 nucleotides or even longer (e.g., Euglenida: *Euglena*). This variation in length makes it very difficult to align eukaryotic rDNA using current alignment software. Benefiting from the results of the comparative studies of the secondary structures of rRNAs, the regions of rDNAs with variable lengths can be conveniently positioned and removed *a priori* from the original rDNA sequence of any species [12]. The improved alignment results of this study greatly facilitated the detection of group-specific nucleotides or indels between the three kingdoms.

In the sequence sampling of this study, both the species diversity and length diversity were taken into account. For the species diversity, the SAR clade ( = Stramenopila+Alveolata+Rhizaria) [14], green plants, fungi, and animals were sampled to the class level, whereas Bacteria, Archaea and the stem groups of Eukaryota were sampled to the order level. For the length diversity, complete or nearly complete sequences with extreme lengths were included.

## Results and Discussion

**Figure 1:**
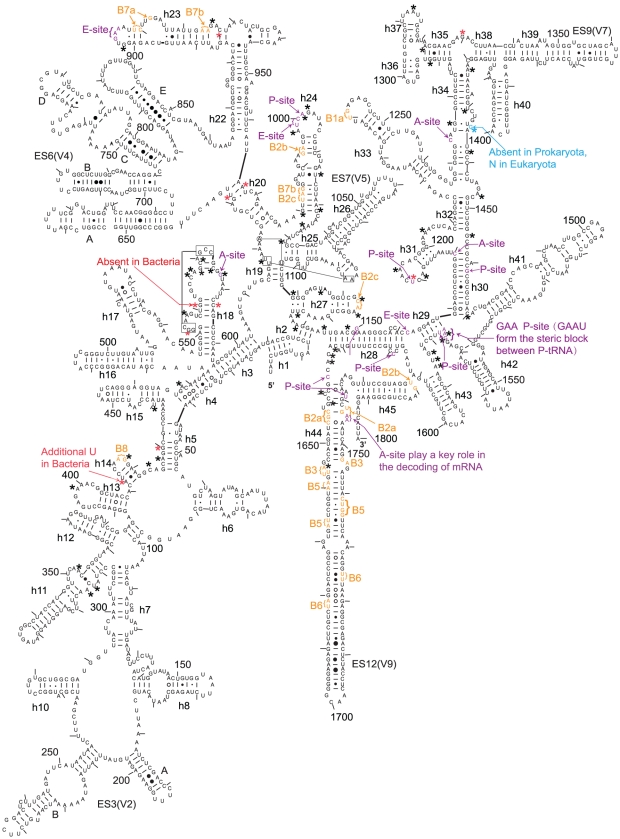
Secondary structure of the 18S rRNA of *Saccharomyces cerevisiae*. The asterisks in different colours mark the nucleotides specifically shared by different organisms, black: cellular organisms; red: archaea and eukaryotes; and blue: eukaryotes. The bases marked in different colours represent those bases that have been determined to have a specific function: orange: in bridging the small and large subunits, purple: in the A, P and E sites.

**Figure 2:**
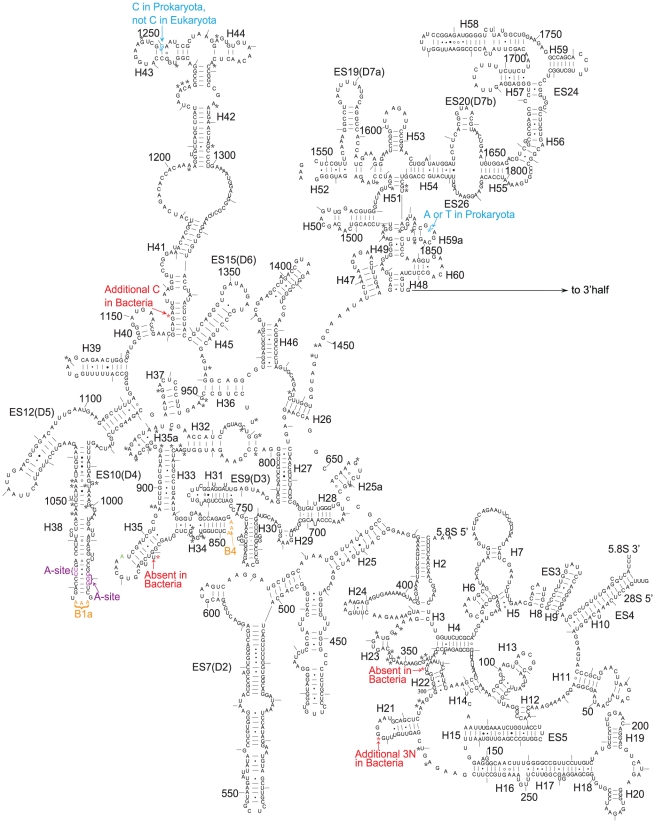
Secondary structure of the 25S rRNA of *Saccharomyces cerevisiae*. The asterisks in different colours mark the nucleotides specifically shared by different organisms, black: cellular organisms; red: archaea and eukaryotes; and blue: archaea and bacteria. The bases marked in different colours represent those bases that have been determined to have a specific function: orange: in bridging the small and large subunits, purple: in the A, P and E sites; and green: in antibiotic resistance sites in the PTC.

**Figure 3:**
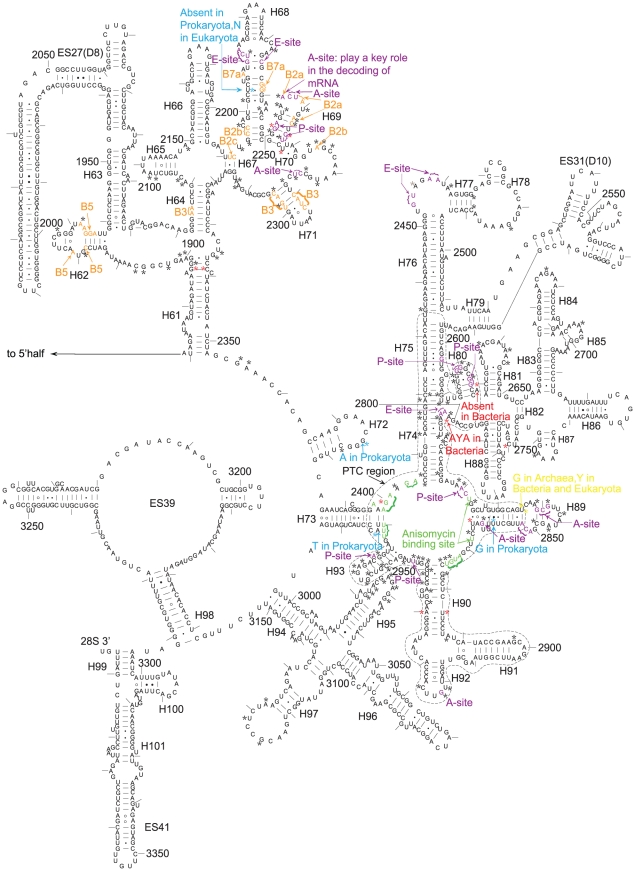
Secondary structure of the 25S rRNA of *Saccharomyces cerevisiae*. The asterisks in different colours mark the nucleotides specifically shared by different organisms, black: cellular organisms; red: archaea and eukaryotes; blue: archaea and bacteria; and yellow: bacteria and eukaryotes. The bases marked in different colours represent those bases that have been determined to have a specific function: orange: in bridging the small and large subunits, purple: in the A, P and E sites; and green: in antibiotic resistance sites in the PTC.

The alignment of the rDNA sequences revealed that ten sites in the 16S–18S rDNA and sixteen sites in the 23S–28S rDNA are shared by all eukaryotes and all archaea but not by bacteria (Table 1 and Figures 1, 2, 3, Files S1, S2, S3, S4, S5, S7, S8, S9, S10, S11, S12, S13, S14, S15). The corresponding nucleotides of *Escherichia coli* and *S. cerevisiae* are listed, and the position numbering of the nucleotide follows that of previous studies on ribosomal structures [15], [16]. Some of these sites are located in known key functional regions of ribosomes [8], [17], [18]. The most noteworthy site is A2058 in *E. coli* (File S12). This base has been shown to be a key site in determining the reaction phenotype to antibiotics in the MLS_B_K family, thus explaining the spectra of drug action [18]–[20]. Only one site is specifically shared by eukaryotes and bacteria (Table 1 and Figure 3). This result explicitly supports the hypothesis that eukaryotic ribosomes, whether larger or smaller than prokaryotic ribosomes, evolved from archaeal ribosomes.

**Table 1:** Ribosomal nucleotides exclusively shared between the three kingdoms.

Base Sharing	Bacteria		Archaea	Eukaryota	
SSU rDNA	124 orders	*E. coli*	21 orders	362 classes/orders	*S. cerevisiae*
A, E	H	T340	-	-	(A412) - (T413)
A, E	Y	T358	G	G	G430
A, E	C	C507	G	D	G553
A, E	-Y	-C514	BG	HR	G561
A, E	R	G537	C	Y	C584
A, E	R	G585	C	Y	T632
A, E	A	A716	C	B	C927
A, E	Y	C756	G	R	A967
A, E	G	G966	T	Y	T1191
A, E	A	A1110	G	G	G1330
B, A	-	(C1203) - (A1204)	(Y)-(A)	N	G1435

LSU rDNA	104 orders	*E. coli*	21 orders	222 classes/orders	*S. cerevisiae*
A, E	VGN	C385G386T387	- - -	- - -	(G281) - - - (G282)
A, E	-	(G411) - (A412)	M	Y	T305
A, E	-	(A739) - (C740)	T	N	T871
A, E	C	C995	(D) - (R)	(N) - (N)	(G1164) - (A1165)
A, E	A	A1665	G	G	G1897
A, E	Y	C1908	G	G	G2251
A, E	R	G1922	C	C	C2265
A, E	C	C1925	T	T	T2268
A, E	T	T1995	C	C	C2338
A, E	A	A2058	G	G	G2400
A, E	-	(T2257) - (C2258)	A	R	A2626
A, E	T	T2390	M	M	C2760
A, E	AYA	A2430T2431A2432	A	A	A2801
A, E	C	C2496	K	T	T2865
A, E	R	A2513	Y	Y	T2882
A, E	Y	T2571	R	A	A2940
B, A	C	C1075	C	D	G1250
B, A	A	A1614	W	C	C1846
B, A	-	(C1843) - (C1844)	(S) - (N)	N	T2203
B, A	A	A2033	A	G	G2375
B, A	G	G2490	G	T	T2859
B, A	T	T2613	T	A	A2982
B, E	Y	C2466	G	Y	T2835

In the first column, A stands for archaea, E stands for eukaryotes, and B stands for Bacteria.

It appears that many clues of the endosymbiotic origin of eukaryotes, which happened approximately 1–2 billion years ago [6], [21], have been eroded, and only a few remain. The limited remaining clues will inevitably lead to some difficulties in determining the complete scheme of the archaeal origin of eukaryotes, especially the formation of the nuclear membrane of eukaryotes [22] and the chimeric properties of eukaryotic genomes [23]. However, the double layers of the nuclear membrane may originate from the endoplasmic reticulum and have different mechanism of origin from those of mitochondria and chloroplasts [1], [6], [24]–[28]. In addition, the chimeric genome can be explained by lateral gene transfer from organelle to nucleus and by fusion between an archaea and a bacterium [23], [29]–[31]. Thus, when conjecturing whether an archaea or a bacterium served as the host cell of endosymbiosis, the former requires fewer hypothesised evolutionary changes.

In addition to the nucleotides specifically shared between archaea and eukaryotes, one site in SSU rDNA and six sites in LSU rDNA were found to be shared by all types of eukaryotes but not by archaea and bacteria (Table 1 and Figures 1, 2, 3, Files S6, S9, S10, S11, S12, S15, S16). These signatures can be viewed as the synapomorphies of Eukaryota and support that all living eukaryotes have a single origin.

**Figure 4:**
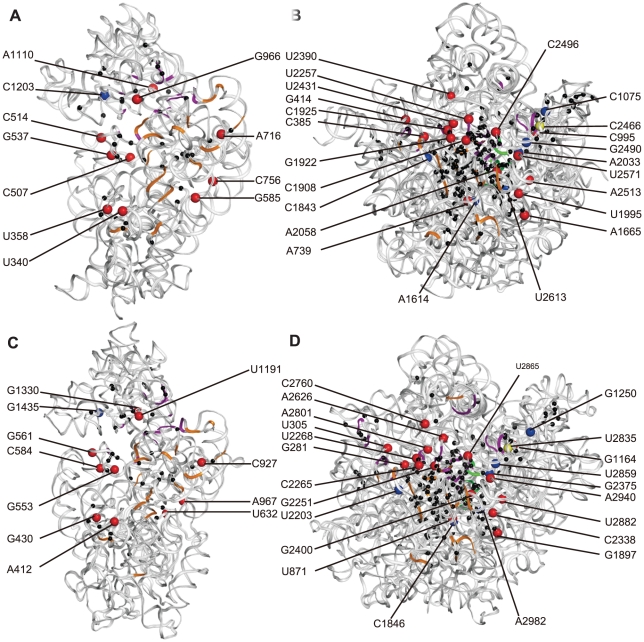
The front view of the tertiary structures of the rRNAs of *E. coli* (A 16S, B 23S) and *S. cerevisiae* (C 18S, D 25S). The accession numbers of these structures in the Protein Data Bank are 2I2U, 2I2V, 3O2Z and 3O58, respectively. The pellets in different colours mark the nucleotides specifically shared by different organisms, black: cellular organisms; red: archaea and eukaryotes; blue: archaea and bacteria; and yellow: bacteria and eukaryotes. The functional sites are shown in different colours; orange: in bridging the small and large subunits, purple: in the A, P and E sites, green: in antibiotic resistance sites in the PTC.

Among the nucleotides that are conserved among all cellular organisms, or in both Archaea and Eukaryota, or just in eukaryotes, some have been determined to be as essential nucleotides in the A, P and E sites or in the bridges between the small and the large subunits of ribosomes [8], [9], [16], [17], [32]–[36] (Figures 1, 2, 3, 4). These nucleotides witness the congruence between the independent results of bioinformatics and structural biology. However, compared to the knowledge of structural biology to date, there exist differences in two areas. The first is that the functions of many conserved or group-specific nucleotides are still unknown. Because these nucleotides may have key functions, they deserve to be paid more attention in structural biology studies in the future. In fact, in the story of A2058 of *E. coli* and homologous G2400 of yeast, structural biologists had suggested that there exist other potential phylogenetic differences involved in drug action [37]–[39]. Given the existence of group-specific indels and substitutions in the peptidyl transferase centre (PTC) region of the 23S–28S subunit, for instance (Figure 3), which is probably the most ancient and key part of the 23S–28S rRNA [20], [40], it is reasonable to postulate that there may exist corresponding group-specific functions. Additionally, as is the case for A2058→G2400, there exist other mutations from bacterial A to eukaryotic G, such as A1110→G1330 in SSU rDNA and A1665→G1897 and A2033→G2375 in LSU rDNA (Table 1, Files S5, S10, S12). The other difference is that some nucleotides that have been identified in structural studies as having important functions are not fully conserved. This result may suggest the structural diversity of these positions among different types of organisms. The results of this work explicitly support the hypothesis that eukaryotic ribosomes evolved from archaeal ribosomes. In addition, all types of eukaryotes, from protists to human beings, have a single origin. The novel conserved or group-specific sites among the three kingdoms provide clear information about the sites that may be critical to the structures and functions of ribosomes. These sites should be experimentally investigated in structural biology studies in the future.

## Materials and Methods

The methods of the annotation of the regions of rDNA with variable lengths followed those of a comparative study of the secondary structure of eukaryotic 18S rRNAs (Xie et al. 2010). In total, 507 taxa for 16–18S rDNA (Table S1) and 347 taxa for 25–28S rDNA were included in this study (Table S2). The alignment was performed using MUSCLE in MEGA5 [41], [42], with a few manual revisions. The information on the conserved nucleotides is based on the program BioEdit [43].

## Supporting Information

Table S1 **Taxon sampling of SSU rDNAs.** There are 124 taxa for bacteria, 21 taxa for archaea and 362 taxa for eukaryotes.
(XLS)

Table S2 **Taxon sampling of LSU rDNAs.** There are 104 taxa for bacteria, 21 taxa for archaea and 222 taxa for eukaryotes.
(XLS)

File S1 **The screen capture 1 of the alignment of SSU (16–18S) rDNAs.** This screen capture is corresponding to the red lines 1–2 of the SSU rDNA part of Table 1. The asterisks in different colors mark the nucleotides specifically shared by different organisms, black: cellular organisms, red: archaea and eukaryotes.
(PDF)

File S2 **The screen capture 2 of the alignment of SSU (16–18S) rDNAs.** This screen capture is corresponding to the red lines 3–6 of the SSU rDNA part of Table 1. The asterisks in different colors mark the nucleotides specifically shared by different organisms, black: cellular organisms, red: archaea and eukaryotes.
(PDF)

File S3 **The screen capture 3 of the alignment of SSU (16–18S) rDNAs.** This screen capture is corresponding to the red lines 7–8 of the SSU rDNA part of Table 1. The asterisks in different colors mark the nucleotides specifically shared by different organisms, black: cellular organisms, red: archaea and eukaryotes.
(PDF)

File S4 **The screen capture 4 of the alignment of SSU (16–18S) rDNAs.** This screen capture is corresponding to the red line 9 of the SSU rDNA part of Table 1. The asterisks in different colors mark the nucleotides specifically shared by different organisms, black: cellular organisms, red: archaea and eukaryotes.
(PDF)

File S5 **The screen capture 5 of the alignment of SSU (16–18S) rDNAs.** This screen capture is corresponding to the red line 10 of the SSU rDNA part of Table 1. The asterisks in different colors mark the nucleotides specifically shared by different organisms, black: cellular organisms, red: archaea and eukaryotes.
(PDF)

File S6 **The screen capture 6 of the alignment of SSU (16–18S) rDNAs.** This screen capture is corresponding to the blue line of the SSU rDNA part of Table 1. The asterisks in different colors mark the nucleotides specifically shared by different organisms, black: cellular organisms, blue: archaea and bacteria.
(PDF)

File S7 **The screen capture 1 of the alignment of LSU (23–28S) rDNAs.** This screen capture is corresponding to the red lines 1–2 of the LSU rDNA part of Table 1. The asterisks in different colors mark the nucleotides specifically shared by different organisms, black: cellular organisms, red: archaea and eukaryotes.
(PDF)

File S8 **The screen capture 2 of the alignment of LSU (23–28S) rDNAs.** This screen capture is corresponding to the red line 3 of the LSU rDNA part of Table 1. The asterisks in different colors mark the nucleotides specifically shared by different organisms, black: cellular organisms, red: archaea and eukaryotes, blue: archaea and bacteria, yellow: bacteria and eukaryotes.
(PDF)

File S9 **The screen capture 3 of the alignment of LSU (23–28S) rDNAs.** This screen capture is corresponding to the red line 4 and the blue line 1 of the LSU rDNA part of Table 1. The asterisks in different colors mark the nucleotides specifically shared by different organisms, black: cellular organisms, red: archaea and eukaryotes, blue: archaea and bacteria.
(PDF)

File S10 **The screen capture 4 of the alignment of LSU (23–28S) rDNAs.** This screen capture is corresponding to the red line 5 and the blue line 2 of the LSU rDNA part of Table 1. The asterisks in different colors mark the nucleotides specifically shared by different organisms, black: cellular organisms, red: archaea and eukaryotes, blue: archaea and bacteria.
(PDF)

File S11 **The screen capture 5 of the alignment of LSU (23–28S) rDNAs.** This screen capture is corresponding to the red lines 6–8 and the blue line 3 of the LSU rDNA part of Table 1. The asterisks in different colors mark the nucleotides specifically shared by different organisms, black: cellular organisms, red: archaea and eukaryotes, blue: archaea and bacteria.
(PDF)

File S12 **The screen capture 6 of the alignment of LSU (23–28S) rDNAs.** This screen capture is corresponding to the red lines 9–10 and the blue line 4 of the LSU rDNA part of Table 1. The asterisks in different colors mark the nucleotides specifically shared by different organisms, black: cellular organisms, red: archaea and eukaryotes, blue: archaea and bacteria.
(PDF)

File S13 **The screen capture 7 of the alignment of LSU (23–28S) rDNAs.** This screen capture is corresponding to the red line 11 of the LSU rDNA part of Table 1. The asterisks in different colors mark the nucleotides specifically shared by different organisms, black: cellular organisms, red: archaea and eukaryotes.
(PDF)

File S14 **The screen capture 8 of the alignment of LSU (23–28S) rDNAs.** This screen capture is corresponding to the red lines 12–13 of the LSU rDNA part of Table 1. The asterisks in different colors mark the nucleotides specifically shared by different organisms, black: cellular organisms, red: archaea and eukaryotes.
(PDF)

File S15 **The screen capture 9 of the alignment of LSU (23–28S) rDNAs.** This screen capture is corresponding to the red lines 14–16, and the blue line 5, and the yellow line of the LSU rDNA part of Table 1. The asterisks in different colors mark the nucleotides specifically shared by different organisms, black: cellular organisms, red: archaea and eukaryotes, blue: archaea and bacteria, yellow: bacteria and eukaryotes.
(PDF)

File S16 **The screen capture 10 of the alignment of LSU (23–28S) rDNAs.** This screen capture is corresponding to the blue line 6 of the LSU rDNA part of Table 1. The asterisks in different colors mark the nucleotides specifically shared by different organisms, black: cellular organisms, blue: archaea and bacteria.
(PDF)
