# Supplementary material for: Potential Key Bases of Ribosomal RNA to Kingdom-Specific Spectra of Antibiotic Susceptibility and the Possible Archaeal Origin of Eukaryotes
Source: PLoS One. 2012 Jan 11;7(1):e29468. doi: 10.1371/journal.pone.0029468 (PMC3256160; doi:10.1371/journal.pone.0029468)

Species/Abbrv

1. ActinobacteriaDQ223228
2. ActinobacteriaNC_002677
3. ActinobacteriaNC_004307
4. ActinobacteriaAB015945
5. ActinobacteriaAB478418
6. ActinobacteriaEF422408
7. ActinobacteriaNC_008148
8. ActinobacteriaNC_013739
9. AquificaeNC_015185
10. BacteroidetesNC_004663
11. BacteroidetesNC_013730
12. BacteroidetesDQ073102
13. BacteroidetesNC_007677
14. ChlorobiNC_002932
15. ChlorobiAB478415
16. CaldisericaAB428365
17. ChlamydiaeNC_002179
18. LentisphaeraeAY390429
19. LentisphaeraeAF275917
20. VerrucomicrobiaNC_010571
21. VerrucomicrobiaNC_014008
22. VerrucomicrobiaY07576
23. VerrucomicrobiaDQ351738
24. VerrucomicrobiaNC_010794
25. ChloroflexiAB046413
26. ChloroflexiAB067647
27. ChloroflexiAF421756
28. ChloroflexiAF039293
29. ChloroflexiNC_007356
30. ChloroflexiAB510917
31. ChloroflexiAB547913
32. ChloroflexiAJ420142
33. ChloroflexiM34115
34. ChrysiogenetesX81319
35. CyanobacteriaNC_007776
36. CyanobacteriaNC_005125
37. CyanobacteriaNC_003272
38. CyanobacteriaNC_008312
39. CyanobacteriaDQ293994
40. CyanobacteriaNC_007335
41. CyanobacteriaX68780
42. CyanobacteriaNC_009925
43. DeferribacteresAB189456
44. Deinococcus-ThermusNC_008025
45. Deinococcus-ThermusNC_006461
46. DictyoglomiX69194
47. ElusimicrobiaNC_010644
48. ElusimicrobiaNS_000191
49. AcidobacteriaNC_014963
50. AcidobacteriaAB303221
51. AcidobacteriaX77215
52. AcidobacteriaAY234728
53. AcidobacteriaNC_008009
54. FibrobacteresAB252949
55. FirmicutesNC_004193
56. FirmicutesNC_005362
57. FirmicutesNC_007907
58. FirmicutesAY965613
59. FirmicutesNC_007644
60. FirmicutesAB034200
61. FirmicutesNC_013520
62. FirmicutesDQ095862
63. FusobacteriaNC_003454
64. GemmatimonadetesDQ453807
65. NitrospiraeX82559
66. PlanctomycetesAB447464
67. PlanctomycetesNC_005027
68. ProteobacteriaNC_002696
69. ProteobacteriaAM749667
70. ProteobacteriaDQ167245
71. ProteobacteriaDQ889871
72. ProteobacteriaAF544015
73. ProteobacteriaNC_008254
74. ProteobacteriaNC_007802
75. ProteobacteriaNC_008343
76. ProteobacteriaNC_006833
77. ProteobacteriaDQ219355
78. ProteobacteriaNC_007722
79. ProteobacteriaNC_003295
80. ProteobacteriaDQ386263
81. ProteobacteriaNC_014394
82. ProteobacteriaNC_007404
83. ProteobacteriaNC_007947
84. ProteobacteriaNC_002946
85. ProteobacteriaNC_004757
86. ProteobacteriaAF177426
87. ProteobacteriaNC_007298
88. ProteobacteriaNC_
89. ProteobacteriaNC_014365
90. ProteobacteriaNC_006138
91. ProteobacteriaNC_007519
92. ProteobacteriaX72768
93. ProteobacteriaNC_007498
94. ProteobacteriaNC_007760
95. ProteobacteriaNC_007759
96. ProteobacteriaNC_007575
97. ProteobacteriaAF357197
98. ProteobacteriaDQ909079
99. ProteobacteriaDQ207728
100. ProteobacteriaNC_006512
101. ProteobacteriaM35016
102. ProteobacteriaNC_007484
103. ProteobacteriaNC_008253
104. ProteobacteriaNC_006369
105. ProteobacteriaNC_002977
106. ProteobacteriaNC_008260
107. ProteobacteriaNC_002663
108. ProteobacteriaNC_004129
109. ProteobacteriaGU452538
110. ProteobacteriaNC_008245
111. ProteobacteriaNC_006370
112. ProteobacteriaNC_007705
113. ProteobacteriaEF493243
114. ProteobacteriaNC_008576
115. SpirochaetesNC_000919
116. TenericutesNC_005303
117. TenericutesAB055910
118. TenericutesNC_006055
119. TenericutesEF999972
120. TenericutesNC_006360
121. ThermodesulfobacteriaAY082369
122. ThermotogaeNC_000853
123. unclassifiedBacNC_013525
124. unclassifiedBacNS_000195
125. CrenarchaeotaNC_014374
126. CrenarchaeotaBA000002
127. CrenarchaeotaEF552404
128. CrenarchaeotaNC_003106
129. CrenarchaeotaNC_003364
130. EuryarchaeotaNC_000917
131. EuryarchaeotaNC_002607
132. EuryarchaeotaNC_000916
133. EuryarchaeotaNC_005791
134. EuryarchaeotaNC_013665
135. EuryarchaeotaNC_007796
136. EuryarchaeotaNC_003552
137. EuryarchaeotaNC_003551
138. EuryarchaeotaNC_000868
139. EuryarchaeotaNC_002578
140. EuryarchaeotaNC_013926
141. EuryarchaeotaNC_009464
142. KorarchaeotaDQ465911
143. NanoarchaeotaNC_005213
144. ThaumarchaeotaNC_014820
145. ThaumarchaeotaNC_010085
146. AmoebozoaAY769863
147. AmoebozoaAF149916
148. AmoebozoaL23799
149. AmoebozoaAY176047
150. AmoebozoaDQ122380
151. AmoebozoaAY775130
152. AmoebozoaAM168079
153. AmoebozoaAF411290
154. AmoebozoaAM231291
155. AmoebozoaFJ766469
156. AmoebozoaAF280078
157. AmoebozoaAY848964
158. AmoebozoaEU137741
159. ApusozoaAY050181
160. CentroheliozoaAF534708
161. CryptophytaL28812
162. CryptophytaAJ010592
163. EuglenozoaAY425012
164. EuglenozoaAY014998
165. EuglenozoaAF386644
166. EuglenozoaAF283307
167. EuglenozoaAJ532396
168. EuglenozoaAY425008
169. EuglenozoaAF110418
170. EuglenozoaAF403160
171. EuglenozoaAC012647
172. EuglenozoaHM004354
173. FornicataGU827589
174. FornicataDQ812526
175. FornicataGU827595
176. FornicataGU827592
177. FornicataGU827604
178. FornicataEU009463
179. GlaucocystophyceaeX68483
180. GlaucocystophyceaeAJ007275
181. GlaucocystophyceaeX70803
182. GlaucocystophyceaeX81901
183. HaptophyceaeAJ246263
184. HaptophyceaeAB183608
185. HaptophyceaeAJ246266
186. HaptophyceaeL34669
187. HaptophyceaeAJ278035
188. HaptophyceaeAM490994
189. HaptophyceaeAM490985
190. HaptophyceaeAB250785
191. HeteroloboseaAF011458
192. HeteroloboseaAF338418
193. JakobidaeAY965870
194. KatablepharidophytaDQ980480
195. MalawimonadidaeAY117420
196. OxymonadidaAY831435
197. ParabasalideaAB458854
198. ParabasalideaGQ254640
199. ParabasalideaAF076958
200. ParabasalideaAB183881
201. ParabasalideaAF821962
202. ParabasalideaU17512
203. ParabasalideaM81842
204. RhodophytaAF342745
205. RhodophytaAF087125
206. RhodophytaAY617133
207. RhodophytaL19345
208. RhodophytaAB183619
209. RhodophytaAF342750
210. ApicomplexaNC_004325
211. ApicomplexaAF017121
212. ApicomplexaDQ174732
213. ApicomplexaDQ093793
214. ChromeridaDQ174731
215. CiliophoraAF145353
216. CiliophoraAF060452
217. CiliophoraAJ292526
218. CiliophoraX65150
219. CiliophoraDQ487201
220. CiliophoraAY332721
221. CiliophoraDQ487194
222. CiliophoraAJ310489
223. CiliophoraAJ277877
224. CiliophoraU24248
225. DinophyceaeAF069516
226. EllobiopsidaeAY340590
227. PerkinseaAF530535
228. VoromonasAF280076
229. RhizariaAB178580
230. RhizariaAF534712
231. RhizariaAY305009
232. ForaminiferaX86093
233. ForaminiferaEU672990
234. ForaminiferaAJ404295
235. ForaminiferaDQ408650
236. ForaminiferaAJ318224
237. ForaminiferaEU649778
238. GromiidaeAJ457811
239. HaplosporidiaDQ219484
240. ParadiniumEU189032
241. PolycystineaAY266295
242. SticholoncheAY268045
243. ActinophryidaeAY305011
244. BacillariophytaAJ867277
245. BacillariophytaX85387
246. BacillariophytaAJ810854
247. BacillariophytaAY485505
248. BacillariophytaAY485501
249. BicosoecidaAF315604
250. BlastocystisAY266468
251. BolidophyceaeAF123595
252. ChrysomerophyceaeU78034
253. ChrysophyceaeGU220392
254. DevelopayellaU37107
255. DictyochophyceaeU14388
256. EustigmatophyceaeAF067956
257. HyphochytriomycetesAF163295
258. LabyrinthulidaAF304465
259. OikomonadaceaeAY520450
260. OomycetesAB287418
261. OomycetesAY744947
262. PelagophyceaeU40258
263. PhaeothamniophyceaeAB365207
264. PicobiliphytesEF050072
265. PinguiophyceaeAB058925
266. PlacidideaAB061218
267. AurearenophyceaeAB365192
268. PhaeophyceaeAB087135
269. XanthophyceaeU41646
270. RaphidophyceaeAB217626
271. SlopalinidaAF141969
272. StramenopilaU73228
273. unclassified_BreviataAF153206
274. unclassified_CollodictyonidaeA
275. unclassified_CryothecomonasAF2
276. unclassified_DimorphaEF455769
277. unclassified_MetromonasAY62025
278. unclassified_MikrocytosHM56306
279. unclassified_MulticiliaAY26803
280. unclassified_PalpitomonasAB508
281. unclassified_ParamonasAY520452
282. unclassified_PhaeomonasAB04220
283. unclassified_ProleptomonasAF41
284. unclassified_PseudodidymiumAY2
285. unclassified_ReticulamoebaAY26
286. unclassified_TelonemiaFJ537314
287. unclassified_TelonemidaAJ56477
288. unclassified_TrimastixAF24490
289. ViridiplantaeX91267
290. ViridiplantaeDQ025753
291. ViridiplantaeAB219527
292. ViridiplantaeZ47997
293. ViridiplantaeM95612
294. ViridiplantaeX75520
295. ViridiplantaeAJ250109
296. ViridiplantaeAF352221
297. ViridiplantaeX70704
298. ViridiplantaeX68825
299. ViridiplantaeX80984
300. ViridiplantaeDQ497432
301. ViridiplantaeAJ269686
302. ViridiplantaeY11370
303. ViridiplantaeX99750
304. ViridiplantaeAJ275005
305. ViridiplantaeX74114
306. ViridiplantaeAF228668
307. ViridiplantaeX80982
308. ViridiplantaeY17604
309. ViridiplantaeY19006
310. ViridiplantaeAY688724
311. ViridiplantaeX80987
312. ViridiplantaeX83521
313. ViridiplantaeX81964
314. ViridiplantaeX78890
315. ViridiplantaeD85301
316. ViridiplantaeAF313565
317. ViridiplantaeEF024047
318. ViridiplantaeX81963
319. ViridiplantaeAY544988
320. ViridiplantaeM20017
321. ViridiplantaeD16448
322. ViridiplantaeL24045
323. ViridiplantaeAF206925
324. ViridiplantaeAF197582
325. ViridiplantaeAF168884
326. ViridiplantaeDQ472350
327. ViridiplantaeX16077
328. FungiDQ883706
329. FungiGU214659
330. FungiAB033529
331. FungiAY544697
332. FungiAJ421689
333. FungiDQ832332
334. FungiAY762621
335. FungiAB003949
336. FungiAF006307
337. FungiU42663
338. FungiZ73326
339. FungiZ27393
340. FungiAB266392
341. FungiZ19578
342. FungiAJ495834
343. FungiFJ869172
344. FungiAY771610
345. FungiAY863105
346. FungiD66881
347. FungiDQ198796
348. FungiAB035721
349. FungiHM371376
350. FungiD14163
351. FungiDQ831029
352. FungiDQ785786
353. FungiDQ846895
354. FungiAY635842
355. FungiDQ536473
356. FungiY17504
357. FungiAB047303
358. FungiDQ536481
359. FungiU35394
360. FungiAB287982
361. FungiEF623861
362. FungiAY603958
363. FungiAB016009
364. Fungi_MicrosporidiaAF033197
365. ChoanoflagellidaAF100941
366. CorallochytriumL42528
367. EccrinalesAY336701
368. FonticulaFJ816018
369. CapsasporaAF349564
370. DermocystidaAF533946
371. IchthyophonidaU25637
372. MinisteriaAF271998
373. NucleariidaeAF349563
374. CnidariaAY026382
375. CnidariaAF358106
376. CnidariaAB231884
377. CnidariaAY039208
378. CtenophoraAF293687
379. CtenophoraAF100944
380. PoriferaAF182190
381. PoriferaAY734444
382. PoriferaAF207844
383. PlacozoaZ22783
384. MyxozoaU70623
385. MyxozoaAM410773
386. AcoelaAF329178
387. Mesozoa_OrthonectidaX97158
388. Mesozoa_RhombozoaX97157
389. MyzostomidaAY210446
390. NemertodermatidaAF051328
391. PlatyhelminthesAJ243681
392. PlatyhelminthesDQ157224
393. PlatyhelminthesAJ228795
394. PlatyhelminthesAJ287562
395. PlatyhelminthesAF013149
396. GastrotrichaAY963687
397. GastrotrichaAY218102
398. GnathostomulidaAY218110
399. GnathostomulidaDQ079929
400. BryozoaAY210443
401. BryozoaU12649
402. BryozoaAY210444
403. CycliophoraAY218107
404. EntoproctaAY210442
405. EntoproctaAY218100
406. EntoproctaU36273
407. MicrognathozoaAY218108
408. RotiferaDQ089736
409. RotiferaU49911
410. RotiferaDQ089737
411. AcanthocephalaAF064817
412. AcanthocephalaAF001842
413. AcanthocephalaAF092433
414. AcanthocephalaAF388660
415. MolluscaX91977
416. MolluscaAF120539
417. MolluscaAY557476
418. MolluscaAY145366
419. MolluscaAF120520
420. MolluscaFJ445774
421. MolluscaX91975
422. MolluscaAF490605
423. NemerteaAY145368
424. NemerteaU29494
425. BrachiopodaDQ279934
426. BrachiopodaX81631
427. PhoronidaAY210450
428. BrachiopodaAF025939
429. AnnelidaZ83756
430. AnnelidaZ83752
431. AnnelidaZ83750
432. AnnelidaAY838853
433. AnnelidaAY708527
434. EchiuraAY210441
435. EchiuraAF342805
436. SipunculaX79874
437. SipunculaAF448166
438. ChaetognathaAY922316
439. ChaetognathaDQ351888
440. ChaetognathaDQ351884
441. KinorhynchaU67997
442. PriapulidaX87984
443. PriapulidaAY746984
444. Metazoa_LoriciferaAY746986
445. NematomorphaAF421771
446. NematomorphaAF421767
447. NematodaX03680
448. NematodaDQ103704
449. NematodaAY851265
450. OnychophoraU49910
451. TardigradaU49909
452. TardigradaAY582119
453. ArthropodaZ74478
454. ArthropodaL81949
455. ArthropodaAY210808
456. ArthropodaAY288701
457. ArthropodaAY859608
458. ArthropodaDQ399857
459. ArthropodaDQ666177
460. ArthropodaAF144214
461. ArthropodaAF370801
462. ArthropodaAJ287064
463. ArthropodaDQ826573
464. ArthropodaAB076658
465. ArthropodaGU067681
466. CollembolaAY555522
467. DipluraAY596366
468. ProturaAY596355
469. ArchaeognathaAF370788
470. ThysanuraAF370790
471. OdonataAF461247
472. EphemeropteraAY749906
473. PlecopteraU68400
474. OrthopteraAY521877
475. ThysanopteraAY900137
476. PsocopteraAY900135
477. PhthirapteraAY077778
478. HemipteraU06475
479. ColeopteraAY521831
480. HymenopteraAY216699
481. LepidopteraAF423783
482. DipteraM21017
483. StrepsipteraX77784
484. XenoturbellidaAY291292
485. HemichordataAF236799
486. HemichordataAF236798
487. EchinodermataAB084553
488. EchinodermataX97156
489. EchinodermataAF279202
490. EchinodermataAY133478
491. EchinodermataAY275898
492. ChordataAB013014
493. ChordataAF236803
494. ChordataD14366
495. ChordataM97571
496. ChordataM97572
497. ChordataAY859632
498. ChordataAY049813
499. ChordataAF021880
500. ChordataL11288
501. ChordataAF188370
502. ChordataX04025
503. ChordataAY859627
504. ChordataAY802766
505. ChordataAJ311672
506. ChordataAF173612
507. ChordataU13369

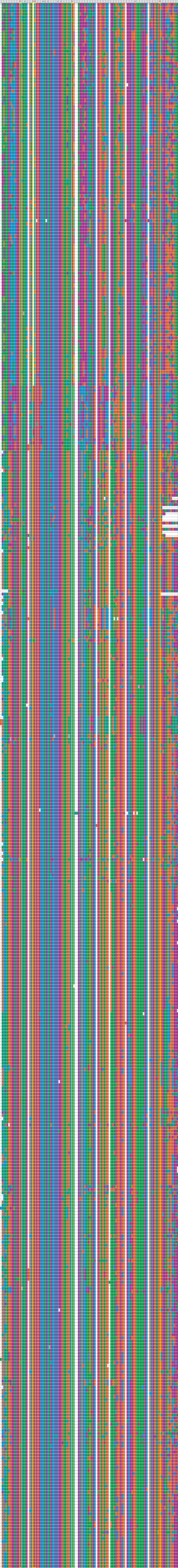

Supplement: File S2 — The screen capture 2 of the alignment of SSU (16–18S) rDNAs. This screen capture is corresponding to the red lines 3–6 of the SSU rDNA part of Table 1. The asterisks in different colors mark the nucleotides specifically shared by different organisms, black: cellular organisms, red: archaea and eukaryotes. (PDF) [file pone.0029468.s004.pdf]
