# Supplementary material for: Potential Key Bases of Ribosomal RNA to Kingdom-Specific Spectra of Antibiotic Susceptibility and the Possible Archaeal Origin of Eukaryotes
Source: PLoS One. 2012 Jan 11;7(1):e29468. doi: 10.1371/journal.pone.0029468 (PMC3256160; doi:10.1371/journal.pone.0029468)
Supplement: File S4 — The screen capture 4 of the alignment of SSU (16–18S) rDNAs. This screen capture is corresponding to the red line 9 of the SSU rDNA part of Table 1. The asterisks in different colors mark the nucleotides specifically shared by different organisms, black: cellular organisms, red: archaea and eukaryotes. (PDF) [file pone.0029468.s006.pdf]

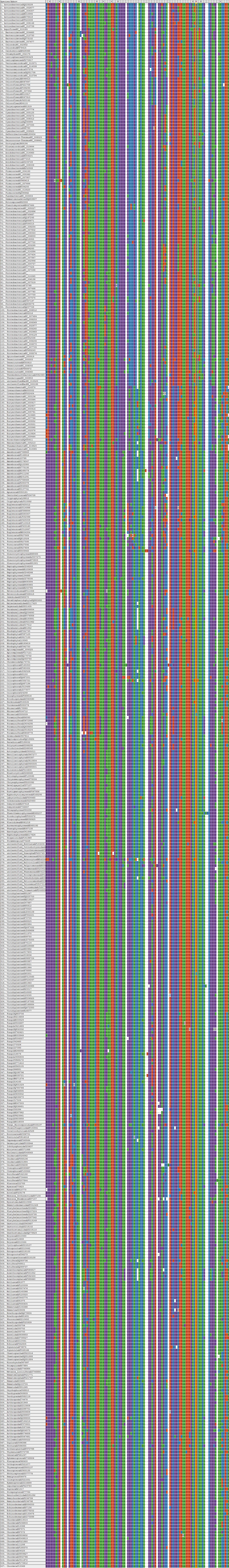
Species/Abbrv
1. ActinobacteriaDQ223228
2. ActinobacteriaNC_002677
3. ActinobacteriaNC_004307
4. ActinobacteriaAB015945
5. ActinobacteriaAB478418
6. ActinobacteriaEF422408
7. ActinobacteriaNC_008148
8. ActinobacteriaNC_013739
9. AquificaeNC_015185
10. BacteroidetesNC_004663
11. BacteroidetesNC_013730
12. BacteroidetesDQ073102
13. BacteroidetesNC_007677
14. ChlorobiNC_002932
15. ChlorobiAB478415
16. CaldisericaAB428365
17. ChlamydiaeNC_002179
18. LentisphaeraeAY390429
19. LentisphaeraeAF275917
20. VerrucomicrobiaNC_010571
21. VerrucomicrobiaNC_014008
22. VerrucomicrobiaY07576
23. VerrucomicrobiaDQ351738
24. VerrucomicrobiaNC_010794
25. ChloroflexiAB046413
26. ChloroflexiAB067647
27. ChloroflexiAF421756
28. ChloroflexiAF039293
29. ChloroflexiNC_007356
30. ChloroflexiAB510917
31. ChloroflexiAB547913
32. ChloroflexiAJ420142
33. ChloroflexiM34115
34. ChrysiogenetesX81319
35. CyanobacteriaNC_007776
36. CyanobacteriaNC_005125
37. CyanobacteriaNC_003272
38. CyanobacteriaNC_008312
39. CyanobacteriaDQ293994
40. CyanobacteriaNC_007335
41. CyanobacteriaX68780
42. CyanobacteriaNC_009925
43. DeferribacteresAB189456
44. Deinococcus-ThermusNC_008025
45. Deinococcus-ThermusNC_006461
46. DictyoglomiX69194
47. ElusimicrobiaNC_010644
48. ElusimicrobiaNS_000191
