# Supplementary material for: Potential Key Bases of Ribosomal RNA to Kingdom-Specific Spectra of Antibiotic Susceptibility and the Possible Archaeal Origin of Eukaryotes
Source: PLoS One. 2012 Jan 11;7(1):e29468. doi: 10.1371/journal.pone.0029468 (PMC3256160; doi:10.1371/journal.pone.0029468)

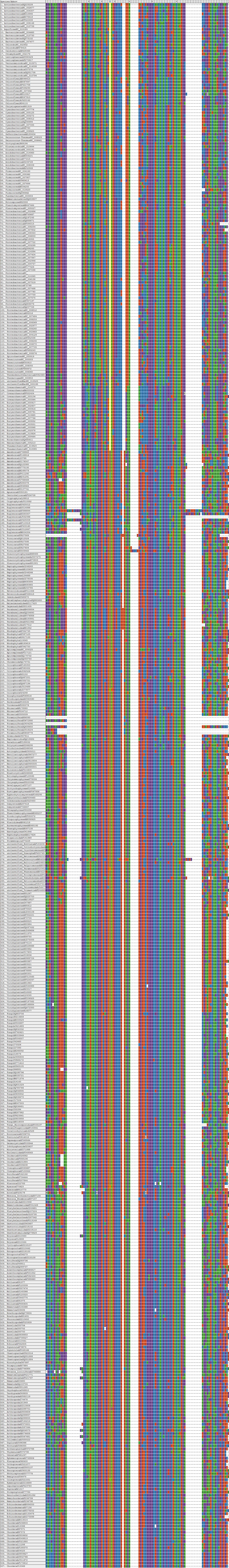

Supplement: File S6 — The screen capture 6 of the alignment of SSU (16–18S) rDNAs. This screen capture is corresponding to the blue line of the SSU rDNA part of Table 1. The asterisks in different colors mark the nucleotides specifically shared by different organisms, black: cellular organisms, blue: archaea and bacteria. (PDF) [file pone.0029468.s008.pdf]
