# Supplementary material for: Potential Key Bases of Ribosomal RNA to Kingdom-Specific Spectra of Antibiotic Susceptibility and the Possible Archaeal Origin of Eukaryotes
Source: PLoS One. 2012 Jan 11;7(1):e29468. doi: 10.1371/journal.pone.0029468 (PMC3256160; doi:10.1371/journal.pone.0029468)
Supplement: File S7 — The screen capture 1 of the alignment of LSU (23–28S) rDNAs. This screen capture is corresponding to the red lines 1–2 of the LSU rDNA part of Table 1. The asterisks in different colors mark the nucleotides specifically shared by different organisms, black: cellular organisms, red: archaea and eukaryotes. (PDF) [file pone.0029468.s009.pdf]

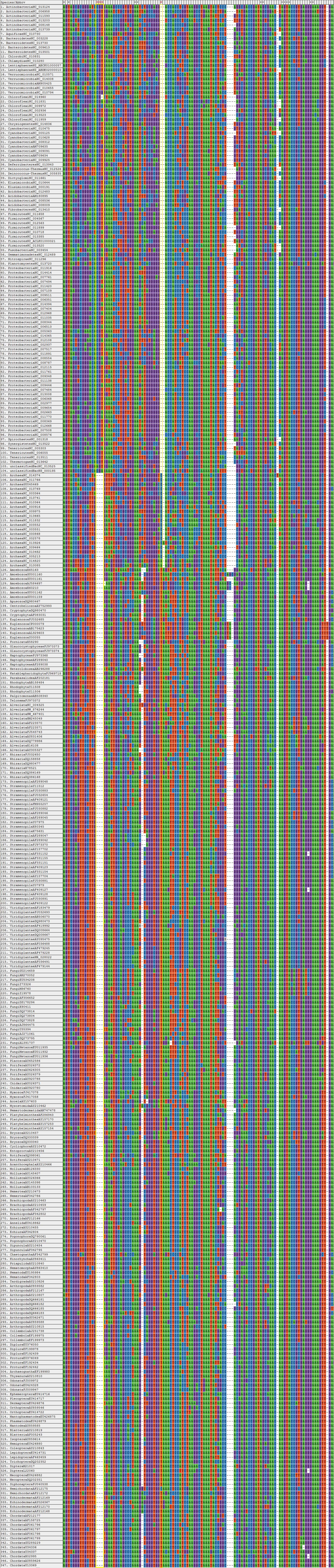
Species/Abbrv
1. ActinobacteriaNC_013124
2. ActinobacteriaNC_006958
3. ActinobacteriaNC_011593
4. ActinobacteriaNC_013203
5. ActinobacteriaNC_008148
6. ActinobacteriaNC_013739
7. AquificaeNC_010730
8. BacteroidetesNC_003228
9. BacteroidetesNC_013730
10. BacteroidetesNC_009613
11. BacteroidetesNC_013501
12. ChlorobiNC_010831
13. ChlamydiaeNC_010280
14. LentisphaeraeNZ_ABCK01000057
15. LentisphaeraeNZ_ABDE02000010
16. VerrucomicrobiaNC_010571
17. VerrucomicrobiaNC_014008
18. VerrucomicrobiaAM905425
19. VerrucomicrobiaNC_010655
20. VerrucomicrobiaNC_010794
21. ChloroflexiNC_014960
22. ChloroflexiNC_011831
23. ChloroflexiNC_009972
24. ChloroflexiNC_002936
25. ChloroflexiNC_013523
26. ChloroflexiNC_011959
27. ChrysiogenetesNC_014836
28. CyanobacteriaNC_010475
29. CyanobacteriaNC_005125
30. CyanobacteriaNC_010628
31. CyanobacteriaNC_008312
32. CyanobacteriaAM709635
33. CyanobacteriaNC_009091
34. CyanobacteriaAM709634
35. CyanobacteriaNC_009925
36. DeferribacteresNC_013943
37. Deinococcus-ThermusNC_012526
38. Deinococcus-ThermusNC_005835
39. DictyoglomiNC_011661
40. ElusimicrobiaNC_010644
41. ElusimicrobiaNS_000191
42. AcidobacteriaNC_012483
43. AcidobacteriaAM180895
44. AcidobacteriaNC_008536
45. AcidobacteriaNC_008009
46. FibrobacteresNC_013410
47. FirmicutesNC_011658
48. FirmicutesNC_004567
49. FirmicutesNC_012563
50. FirmicutesNC_011899
51. FirmicutesNC_010718
52. FirmicutesNC_013385
53. FirmicutesNZ_ACLK01000021
54. FirmicutesNC_013520
55. FusobacteriaNC_003454
56. GemmatimonadetesNC_012489
57. NitrospiraeNC_011296
58. PlanctomycetesNC_013720
59. ProteobacteriaNC_011916
60. ProteobacteriaNC_014414
61. ProteobacteriaNC_007761
62. ProteobacteriaNC_007494
63. ProteobacteriaNC_011420
64. ProteobacteriaNC_007109
65. ProteobacteriaNC_009511
66. ProteobacteriaNC_006351
67. ProteobacteriaNC_014394
68. ProteobacteriaNC_007404
69. ProteobacteriaNC_012968
70. ProteobacteriaNC_011035
71. ProteobacteriaNC_008344
72. ProteobacteriaNC_006513
73. ProteobacteriaNC_005363
74. ProteobacteriaNC_014365
75. ProteobacteriaNC_012108
76. ProteobacteriaNC_002937
77. ProteobacteriaNC_007517
78. ProteobacteriaNC_011891
79. ProteobacteriaNC_008554
80. ProteobacteriaNC_008787
81. ProteobacteriaNC_012115
82. ProteobacteriaNC_011761
83. ProteobacteriaNC_009348
84. ProteobacteriaNC_011138
85. ProteobacteriaNC_009446
86. ProteobacteriaNC_007484
87. ProteobacteriaNC_013008
88. ProteobacteriaNC_006368
89. ProteobacteriaNC_002977
90. ProteobacteriaNC_009654
91. ProteobacteriaNC_002663
92. ProteobacteriaNC_011770
93. ProteobacteriaNC_008369
94. ProteobacteriaNC_012668
95. ProteobacteriaNC_007508
96. ProteobacteriaNC_008576
97. SpirochaetesNC_001318
98. SynergistetesNC_013522
99. TenericutesNC_005303
100. TenericutesNC_006055
101. TenericutesNC_013511
102. ThermotogaeNC_013642
103. unclassifiedBacNC_013525
104. unclassifiedBacNS_000195
105. ArchaeaNC_014374
106. ArchaeaNC_011766
107. ArchaeaGU045469
108. ArchaeaNC_013769
109. ArchaeaNC_003364
110. ArchaeaNC_013741
111. ArchaeaNC_010364
112. ArchaeaNC_000916
113. ArchaeaNC_009975
114. ArchaeaNC_013665
115. ArchaeaNC_011832
116. ArchaeaNC_003552
117. ArchaeaNC_003551
118. ArchaeaNC_000868
119. ArchaeaNC_002578
120. ArchaeaNC_013926
121. ArchaeaNC_009464
122. ArchaeaNC_010482
123. ArchaeaNC_005213
124. ArchaeaNC_014820
125. ArchaeaNC_010085
126. AmoebozoaX65163
127. AmoebozoaGU001160
128. AmoebozoaGU001161
129. AmoebozoaAJ584697
130. AmoebozoaX60210
131. AmoebozoaGU001162
132. AmoebozoaGU001159
133. ApusozoaDQ980467
134. CentroheliozoaAY752993
135. CryptophytaDQ980470
136. CryptophytaAF083031
137. EuglenozoaFJ032685
138. EuglenozoaCP000079
139. EuglenozoaAB175623
140. EuglenozoaAL929603
141. EuglenozoaY00055
142. FornicataX58290
143. GlaucocystophyceaeFJ973373
144. GlaucocystophyceaeFJ973374
145. HaptophyceaeFJ973368
146. HaptophyceaeAF289040
147. HaptophyceaeAF289038
148. HeteroloboseaAB298288
149. KatablepharidophytaFJ969718
150. ParabasalideaAF202181
151. RhodophytaAB158484
152. RhodophytaY11508
153. RhodophytaY11506
154. PalpitomonasAB508340
155. TelonemaFJ973372
156. AlveolataNC_004325
157. AlveolataNW_876244
158. AlveolataNW_667401
159. AlveolataHM245049
160. AlveolataAF223570
161. AlveolataAF223571
162. AlveolataFJ545743
163. AlveolataAY831406
164. AlveolataDQ779986
165. AlveolataX16108
166. AlveolataAY305327
167. RhizariaFJ032650
168. RhizariaDQ158858
169. RhizariaDQ980477
170. RhizariaX78521
171. RhizariaDQ386169
172. RhizariaDQ386168
173. StramenopilaAF289048
174. StramenopilaY11512
175. StramenopilaFJ030883
176. StramenopilaFJ032654
177. StramenopilaAF409121
178. StramenopilaFM955257
179. StramenopilaFJ030882
180. StramenopilaAF289046
181. StramenopilaAF289045
182. StramenopilaY07975
183. StramenopilaX80345
184. StramenopilaX75631
185. StramenopilaAF289047
186. StramenopilaAF289042
187. StramenopilaFJ973370
188. StramenopilaAY157702
189. StramenopilaAF331152
190. StramenopilaAF331155
191. StramenopilaAF331151
192. StramenopilaAF409123
193. StramenopilaAF331154
194. StramenopilaAY157704
195. StramenopilaFJ030881
196. StramenopilaY07979
197. StramenopilaAF409127
198. StramenopilaAF409128
199. StramenopilaFJ030891
200. StramenopilaAF409122
201. ViridiplantaeAF183479
202. ViridiplantaeFJ032693
203. ViridiplantaeAB506070
204. ViridiplantaeDQ980473
205. ViridiplantaeAF419992
206. ViridiplantaeDQ008664
207. ViridiplantaeDQ008667
208. ViridiplantaeAY095475
209. ViridiplantaeAF036488
210. ViridiplantaeAF479245
211. ViridiplantaeAF479228
212. ViridiplantaeNR_028022
213. ViridiplantaeAF036491
214. ViridiplantaeAF479144
215. FungiGU214659
216. FungiAM270052
217. FungiEU534208
218. FungiZ73326
219. FungiM86760
220. FungiZ19578
221. FungiAF356652
222. FungiDS178294
223. FungiX90411
224. FungiDQ273814
225. FungiDQ273804
226. FungiDQ273828
227. FungiAJ864475
228. FungiU35394
229. FungiAJ271061
230. FungiDQ273795
231. FungiAL391737
232. FungiMetazoaEU011935
233. FungiMetazoaEU011932
234. FungiMetazoaEU011936
235. PlacozoaAY652586
236. PoriferaAY026372
237. PoriferaAY626305
238. PoriferaAY026379
239. CnidariaAY920788
240. CnidariaAY026371
241. CnidariaAY920780
242. MyxozoaFJ417078
243. MyxozoaFJ417058
244. AcoelaAY157603
245. MyzostomidaAY210462
246. NemertodermatidaAM747478
247. PlatyhelminthesAF286943
248. PlatyhelminthesAY157170
249. PlatyhelminthesAY157253
250. PlatyhelminthesAY157154
251. BryozoaAY210457
252. BryozoaDQ333339
253. BryozoaDQ333340
254. CycliophoraAY210472
255. EntoproctaAY210456
256. RotiferaGQ398061
257. RotiferaAY210471
258. AcanthocephalaAY210466
259. MolluscaAB126330
260. MolluscaAY145407
261. MolluscaAY026366
262. MolluscaAY145398
263. MolluscaAB103133
264. NemerteaAY210473
265. NemerteaAF342786
266. BrachiopodaAY210463
267. BrachiopodaAY210459
268. BrachiopodaAF342797
269. BrachiopodaAF342802
270. AnnelidaAF212166
271. AnnelidaEU418862
272. EchiuraAY210455
273. EchiuraAF342804
274. PogonophoraDQ790061
275. PogonophoraAY210470
276. SipunculaAY210454
277. SipunculaAF342795
278. ChaetognathaAF342799
279. KinorhynchaAY863411
280. PriapulidaAY210840
281. NematomorphaAY863410
282. NematodaEU195984
283. NematodaAF342803
284. TardigradaAY210826
285. ArthropodaAY859582
286. ArthropodaAF212167
287. ArthropodaAY210807
288. ArthropodaDQ666181
289. ArthropodaDQ666182
290. ArthropodaDQ666185
291. ArthropodaDQ666183
292. ArthropodaGU362671
293. ArthropodaAY859588
294. ArthropodaAY210815
295. CollembolaAJ251738
296. CollembolaEF199975
297. CollembolaEF199973
298. DipluraEU376050
299. DipluraEF199978
300. DipluraEF192439
301. ProturaEU376049
302. ProturaEF192434
303. ProturaEF192442
304. ArchaeognathaEF199980
305. ThysanuraAY210810
306. OdonataFJ009972
307. OdonataEU424328
308. OdonataFJ009947
309. EphemeropteraEU414716
310. PlecopteraEU414717
311. DermapteraEU426876
312. OrthopteraAY859546
313. OrthopteraEU414722
314. MantophasmatodeaEU426875
315. PhasmatodeaEU426879
316. MantodeaAY859585
317. BlatteriaAY210819
318. BlatteriaAF005243
319. IsopteraAY859614
320. HemipteraEU426880
321. ColeopteraAY210843
322. LepidopteraEU414731
323. LepidopteraAF463459
324. TrichopteraDQ202352
325. DipteraM21017
326. DipteraL22060
327. MecopteraEU426882
328. MecopteraDQ202351
329. SiphonapteraFJ040538
330. HemichordataAF212175
331. HemichordataAF212172
332. EchinodermataAF212169
333. EchinodermataAY026367
334. EchinodermataAF212170
335. EchinodermataAF212168
336. ChordataAF212177
337. ChordataAF158725
338. ChordataAF061796
339. ChordataAF061797
340. ChordataAF061798
341. ChordataAF061799
342. ChordataGU289229
343. ChordataU34336
344. ChordataU34337
345. ChordataX02995
346. ChordataAY859626
347. ChordataU13369
