# Supplementary material for: Potential Key Bases of Ribosomal RNA to Kingdom-Specific Spectra of Antibiotic Susceptibility and the Possible Archaeal Origin of Eukaryotes
Source: PLoS One. 2012 Jan 11;7(1):e29468. doi: 10.1371/journal.pone.0029468 (PMC3256160; doi:10.1371/journal.pone.0029468)
Supplement: File S9 — The screen capture 3 of the alignment of LSU (23–28S) rDNAs. This screen capture is corresponding to the red line 4 and the blue line 1 of the LSU rDNA part of Table 1. The asterisks in different colors mark the nucleotides specifically shared by different organisms, black: cellular organisms, red: archaea and eukaryotes, blue: archaea and bacteria. (PDF) [file pone.0029468.s011.pdf]

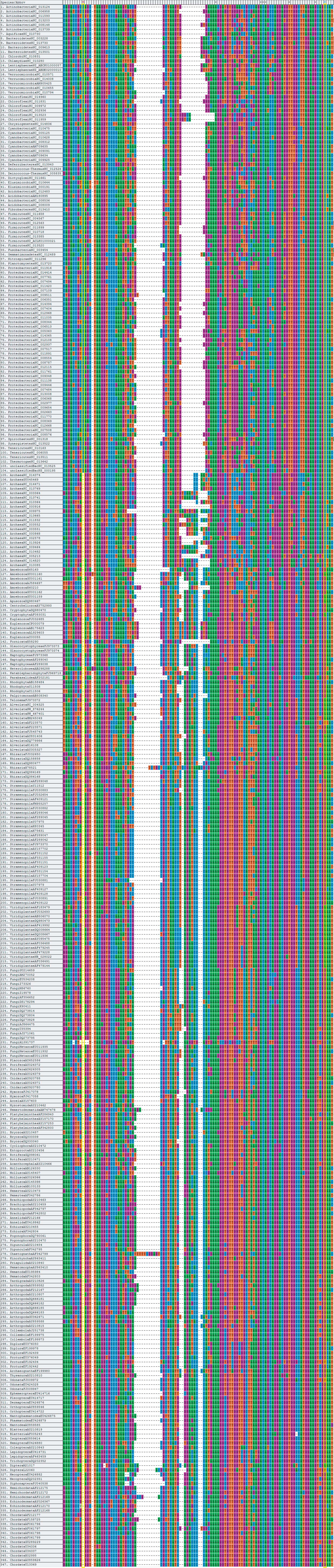
Species/Abbrv
